# Forced anal examinations to ascertain sexual orientation and sexual behavior: An abusive and medically unsound practice

**DOI:** 10.1371/journal.pmed.1002536

**Published:** 2018-03-16

**Authors:** Cody Cichowitz, Leonard Rubenstein, Chris Beyrer

**Affiliations:** 1 Center for Public Health and Human Rights, Department of Epidemiology, Johns Hopkins Bloomberg School of Public Health, Baltimore, Maryland, United States of America; 2 Johns Hopkins School of Medicine, Baltimore, Maryland, United States of America

## Abstract

In an essay, Cody Cichowitz and colleagues discuss approaches to preventing the practice of inappropriate anal examination.

**Summary points**

- It has been reported that, in at least nine countries, forced anal examinations are used to investigate or punish alleged same-sex behavior between consenting men or transgender women.
- In these settings, forced anal examinations are usually performed by healthcare providers at the request of law enforcement officials, and are procedures entirely distinct from those used legitimately in clinical care.
- Forced anal examinations are intended to cause physical and/or emotional pain and offer no potential benefits to the targeted individual, violating key principles of medical ethics and human rights. Such examinations constitute acts of torture under the United Nations Convention against Torture.
- Due to the possibility of coercion, individual physicians may face challenges resisting requests to perform such procedures, although they are abusive, medically unindicated, and yield no helpful information. Professional societies and organizations may be best positioned to oppose states’ attempts to use a medical procedure to oppress a vulnerable population.
- Healthcare providers, professional organizations, and normative agencies, including the World Health Organization, all have an important role to play in bringing about an end to this practice.

In October of 2016, the Tanzanian government forced the closure of internationally supported HIV testing and treatment centers designed to serve key populations, including sex workers and men who have sex with men (MSM) [1]. This controversial decision and the larger ongoing government crackdown on lesbian, gay, bisexual, and transgender (LGBT) communities in Tanzania have garnered the attention of public health officials and international journalists, who have expressed concerns about their health ramifications and associated human rights violations [2–4]. It has been reported that Tanzanian physicians, under the direction of law enforcement, have subjected men accused of same-sex behavior to forced anal examination in order to determine their sexual orientation [4,5]. One victim described being arrested at a birthday celebration and taken to a hospital, where a doctor performed an anal examination in front of a police officer that left the man feeling as if he had been raped [4].

The situation in Tanzania is not unique. Over the past seven years, forced anal examinations have reportedly occurred in at least nine countries, without clinical indication, to aid in the prosecution of alleged same-sex behavior between consenting adult men or transgender women [5,6]. The consequences of successful criminal prosecution may be severe; for example, in Tanzania, homosexuality can be punished by 30 years up to a lifetime in prison [7]. In several countries, forced anal examinations are reported to have been performed by healthcare providers at the request of police or other security operatives and consist of procedures entirely distinct from any examination of the anus, anal canal, or rectum used in clinical care [6].

In clinical care, anal examinations are routinely preformed for specific indications and to answer carefully formulated diagnostic questions. They provide necessary information in the workup and treatment of highly prevalent conditions, including prostate cancer, anal fissures and fistulas, hemorrhoids, and gastrointestinal bleeding. In stark contrast, forced anal exams intentionally inflict significant physical and/or mental pain and suffering at the behest of the state as a form of punishment, meeting the United Nations (UN) definition of torture [8]. During examination, individuals may be stripped, compelled to assume humiliating positions, and examined in front of others, without consent or under coercion. The exam itself may consist of the forceful insertion of fingers, tubes, funnels, or other objects into the anus and rectum and can result in physical and/or emotional trauma [6].

## Bad science and false evidence

The earliest recorded tests purported to detect homosexuality were described by a 19th century French forensic doctor who erroneously claimed that six criteria could be used to identify homosexuality on examination [9]. Despite being published in the 1800s and discredited by medical professionals then and now, the ideas put forth continue to inform states’ practices—namely that examination of the anal sphincter can correctly determine whether or not an individual has participated in consensual receptive anal intercourse [9]. More recently, in Egypt, physicians have attempted to determine this through electromyography, sonography, and manometry of the anal sphincter, pelvic floor, and rectum, respectively [9]. These investigations are methodologically flawed, medically unsubstantiated, and constitute grave ethical violations. The so-called research is often conducted on prisoners or detainees and confers harm with no potential for individual or societal benefit [9].

In 2015, the Office of the United Nations High Commissioner for Human Rights recommended that forced anal examinations be banned worldwide [10]; subsequently, a joint UN statement was released denouncing the practice [11]. In 2016, an independent panel of forensic experts including doctors, psychologists, and psychiatrists issued a statement unequivocally refuting all claims that anal examination can identify sexual practices [12]. The statement discredited the most commonly cited reason for conducting exams—to determine anal sphincter tone. The panel highlighted the fact that there are no standardized ways of determining anal tone with digital rectal examination, that there are high levels of normal variability within the population, and that numerous conditions may affect anal tone, independent of any practiced behaviors. Additionally, the panel described some of the negative consequences of the exams, including pain; trauma to the anus, anal canal, or rectum; humiliation; and shame [12]. It is likely that forced anal examinations produce a profile of psychiatric morbidity similar to rape or sexual harassment, both of which are associated with a heightened risk of suicidality and post-traumatic stress disorder [12,13].

## Ethical ramifications

Central to the practice of medicine is the Hippocratic Oath, to first do no harm. In the case of forced anal examination, a routine clinical practice is transformed into a direct punishment, conducted with the intent to harm members of the LGBT community. A malicious procedure is employed without medical indication or forensic justification. Forced anal examinations require physicians to disregard their obligations to exercise independent professional judgment and to base their interventions on sound clinical knowledge. These professional obligations are especially important when an individual is in state custody, their choices are constrained, and the risks of harm are great.

Forced anal examinations constitute acts of torture, which are defined by the UN as acts that intentionally inflict severe pain or suffering to punish a victim “for any reason based on any kind of discrimination” [8]. Torture itself violates the ethical and professional principles central to medicine, and clinicians’ involvement in torture and cruel, inhumane treatment is prohibited by the World Medical Association’s (WMA) Declaration of Tokyo and the UN’s Principles of Medical Ethics applicable to places of detention [14,15]. The UN’s Principles of Medical Ethics accordingly state that “it is a contravention of medical ethics for health personnel, particularly physicians, to be involved in any professional relationship with prisoners or detainees the purpose of which is not solely to evaluate, protect or improve their physical and mental health” [14]. Recognizing this, the WMA passed a resolution denouncing forced anal examinations to substantiate same-sex activity and called for an immediate stop to medically useless exams that are outside accepted standards of practice [16].

Healthcare workers may face challenges in resisting state-sponsored medical ethics and human rights violations [17,18], particularly when states are using providers to achieve political ends and oppress a vulnerable population. For this reason, the importance of the recent WMA resolution cannot be overstated as a means to resist efforts by governments to coerce health professionals to perform these examinations.

States’ utilization of medical professionals to torture marginalized populations perpetuates health disparities, increases stigma, undermines advances in access to care, and erodes the trust foundational to the doctor–patient relationship. Forced anal examinations (and even the threat of their occurrence) may also undermine the effectiveness of community-based LGBT organizations; individuals’ willingness to disclose their sexual orientation and behaviors to their healthcare providers; and ongoing efforts to improve HIV diagnosis, treatment, and care for MSM.

## Scope of the problem and recent progress

Over the past seven years, several countries have reportedly used the findings of forced anal examinations as official evidence in legal proceedings [6]. In Egypt and Tunisia, forensic medical specialists submit a report describing the results of the exam as evidence to be used in court, and similar procedures have been used in Cameroon, Zambia, and Lebanon. In Uganda, Kenya, Tanzania, and Turkmenistan, exams may be used in conjunction with an investigation or conducted unofficially to harm or punish suspected individuals [6]. Little is known about the true prevalence of this practice, but the fact that it is codified in legal systems, used in multiple countries, and is the subject of both human rights and news reports suggests an immediate need for advocacy and intervention.

Encouragingly, three of the nine countries reported to have used forced anal examination have made significant progress toward ending their use [5,6,19]. In each country, activists, human rights workers, and healthcare providers have partnered together to initiate change. In Lebanon, advocates successfully built public support and pressured the Lebanese Order of Physicians and the Ministry of Justice to promulgate guidelines prohibiting the use of forced anal exams [5,19]. In Kenya, the Kenyan Medical Association released a statement condemning any and all types of forced anal examination despite directives from law enforcement officials [19]. In Tunisia, the government banned the practice after physicians repeatedly refused to administer the exam and human rights groups advocated for policy change [5,19].

## Time to act

These three countries and the movements within them offer hope and tangible examples for how citizens, healthcare professionals, and allied organizations can stand together. Individual providers asked to perform forced anal examinations should resist the practice whenever possible, even when they themselves are being coerced or intimidated to conduct these examinations. Professional organizations and normative agencies may be better positioned to circumvent the challenges facing individuals. These organizations often have great influence on local governments and ministries of health and regularly set standards of practice. They should use their power and influence to more effectively oppose this practice and promote adherence to professional and ethical codes.

We believe that the global medical community should actively support individuals targeted by forced anal examinations and partner with colleagues and professional organizations in countries that perform these exams to end their use. Normative agencies, including the World Health Organization, should condemn forced anal examination and call for its global ban, publicly declaring that these exams amount to torture and cruel, inhuman, and degrading treatment. It is time to unite in action and bring about an end to this egregious practice.

## Abbreviations

LGBT: lesbian, gay, bisexual, and transgender
MSM: men who have sex with men
UN: United Nations
WMA: World Medical Association

## References

[1] Mwalimu U. Statement by the Minster for Health, Community Development, Gender, Elderly and Children, HON. Ummy Mwalimu on key populations HIV services in Tanzania. The United Republic of Tanzania: Ministry of Health, Community Development, Gender, Elderly, and Children. 27 10 2016 [Cited 27 January 2018]. Available from: https://m.facebook.com/afyatz/posts/1230922863594845.

[2] Sieff K. Tanzania suspends U.S.- funded AIDS programs in a new crackdown on gays The Washington Post 23 11 2016 [Cited 27 January 2018]. Available from: https://www.washingtonpost.com/world/africa/tanzania-suspends-us-funded-aids-programs-in-a-new-crackdown-on-gays/2016/11/23/ec6ced6e-ab5c-11e6-8f19-21a1c65d2043_story.html.

[3] Fallon A. People With HIV Are Panicking Due To Tanzania's Crackdown On Gays NPR—Goats and Soda 15 3 2017 [Cited 27 January 2018]. Available from: https://www.npr.org/sections/goatsandsoda/2017/03/15/518155924/people-with-hiv-are-panicking-due-to-tanzanias-crackdown-on-gays.

[4] Honan E. How Tanzania Is Cracking Down On LGBT People—And Getting Away With It Buzzfeed 8 4 2017 [Cited 27 January 2018]. Available from: https://www.buzzfeed.com/edithhonan/how-tanzania-is-cracking-down-on-lgbt-people-and-getting?utm_term=.le6rnV4Oy#.lwOQrGP0b.

[5] Human Rights Watch. Global Medical Body Condemns Forced Anal Exams Human Rights Watch 17 10 2017 [Cited 27 January 2018]. Available from: https://www.hrw.org/news/2017/10/17/global-medical-body-condemns-forced-anal-exams.

[6] Human Rights Watch. Dignity debased: Forced anal examinations in homosexuality prosecutions Human Rights Watch 12 7 2016 [Cited 27 January 2018]. Available from: https://www.hrw.org/report/2016/07/12/dignity-debased/forced-anal-examinations-homosexuality-prosecutions.

[7] Moen K, Aggleton P, Leshabari MT, Middelthon A-L. Same-Sex Practicing Men in Tanzania from 1860 to 2010. Arch Sex Behav. 2014 4 22;43(6):1065–82. doi: 10.1007/s10508-014-0286-2 24752788 10.1007/s10508-014-0286-2

[8] United Nations. Convention against torture and other cruel, inhuman or degrading treatment or punishment United Nations 10 12 1984 [Cited 27 January 2018]. Available from: http://www.ohchr.org/Documents/ProfessionalInterest/cat.pdf.

[9] Long S. When Doctors Torture: The Anus and the State in Egypt and Beyond. Health Hum Rights. 2008 3 6;7:116–40.

[10] Human Rights Council. Discrimination and violence against individuals based on their sexual orientation and gender identity United Nations 4 5 2015 [Cited 27 January 2018]. Available from: https://undocs.org/A/HRC/29/23.

[11] United Nations. Ending violence and discrimination against lesbian, gay, bisexual, transgender and intersex people. 29 Sep 2015. [Cited 27 January 2018]. Available from: http://www.ohchr.org/Documents/Issues/Discrimination/Joint_LGBTI_Statement_ENG.PDF.

[12] International Forensic Expert Group. Statement on anal examinations in cases of alleged homosexuality. Torture. 2016;26(2):85–91. 27858782

[13] Dworkin ER, Menon SV, Bystrynski J, Allen NE. Sexual assault victimization and psychopathology: A review and meta-analysis. Clin Psychol Rev. 2017;56:65–81. doi: 10.1016/j.cpr.2017.06.002 28689071 10.1016/j.cpr.2017.06.002 PMC5576571

[14] United Nations. Principles of medical ethics relevant to the role of health personnel United Nations 18 12 1982 [Cited 27 January 2018]. Available from: http://www.ohchr.org/EN/ProfessionalInterest/Pages/MedicalEthics.aspx.

[15] World Medical Association. Declaration of Tokyo World Medical Association 10 1975 [Cited 27 January 2018]. Available from: https://www.wma.net/policies-post/wma-declaration-of-tokyo-guidelines-for-physicians-concerning-torture-and-other-cruel-inhuman-or-degrading-treatment-or-punishment-in-relation-to-detention-and-imprisonment/.

[16] World Medical Association. Resolution on prohibition of forced anal examinations to substantiate same-sex sexual activity World Medical Association 10 2017 [Cited 27 January 2018]. Available from: https://www.wma.net/policies-post/wma-resolution-on-prohibition-of-forced-anal-examinations-to-substantiate-same-sex-sexual-activity/.

[17] Mateen FJ, Rubenstein LS. Government policies in violation of human rights as a barrier to professionalism. JAMA. 2011 8 3;306(5):541–2. doi: 10.1001/jama.2011.1082 21813432 10.1001/jama.2011.1082

[18] Pont J, Stöver H, Wolff H. Dual loyalty in prison health care. Am J Public Health. 2012 3;102(3):475–80. doi: 10.2105/AJPH.2011.300374 22390510 10.2105/AJPH.2011.300374 PMC3487660

[19] Ghoshal N. Beginning of end for forced anal exams Human Rights Watch 26 9 2017 [Cited 27 January 2018]. Available from: https://www.hrw.org/news/2017/09/26/beginning-end-forced-anal-exams.

